# No difference between mobile and fixed bearing in primary total knee arthroplasty: a meta-analysis

**DOI:** 10.1007/s00167-022-07065-5

**Published:** 2022-07-21

**Authors:** Filippo Migliorini, Nicola Maffulli, Francesco Cuozzo, Marco Pilone, Karen Elsner, Jörg Eschweiler

**Affiliations:** 1 Department of Orthopaedic, Trauma, and Reconstructive Surgery, RWTH University Hospital, Pauwelsstraße 30, 52074 Aachen, Germany; 2 Department of Medicine, Surgery and Dentistry, University of Salerno, 84081 Baronissi, SA Italy; 3 School of Pharmacy and Bioengineering, Keele University Faculty of Medicine, ST4 7QB Stoke-on-Trent, England; 4 Barts and the London School of Medicine and Dentistry, Centre for Sports and Exercise Medicine, Mile End Hospital, Queen Mary University of London, E1 4DG London, England

**Keywords:** Total knee arthroplasty, Mobile bearing, Fixed bearing

## Abstract

**Purpose:**

Both mobile (MB) and fixed (FB) bearing implants are routinely used for total knee arthroplasty (TKA). This meta-analysis compared MB *versus* FB for TKA in terms of implant positioning, joint function, patient reported outcome measures (PROMs), and complications. It was hypothesised that MB performs better than FB implants in primary TKA.

**Methods:**

This meta-analysis was conducted according to the 2020 PRISMA statement. In February 2022, the following databases were accessed: Pubmed, Web of Science, Google Scholar, Embase. All the randomized clinical trials (RCTs) comparing mobile *versus* fixed bearing for primary TKA were considered.

**Results:**

Data from 74 RCTs (11,116 procedures) were retrieved. The mean follow-up was 58.8 (7.5 to 315.6) months. The MB group demonstrated greater range of motion (ROM) (*P* = 0.02), Knee Society Score (KSS) score (*P* < 0.0001), and rate of deep infections (*P* = 0.02). No difference was found in implant positioning: tibial slope, delta angle, alpha femoral component angle, gamma femoral component angle, beta tibial component angle, tibiofemoral alignment angle, posterior condylar offset, radiolucent lines. No difference was found in duration of the surgical procedure. No difference was found in the following PROMs: Oxford Knee Score (OKS), Western Ontario and McMaster Universities Osteoarthritis Index (WOMAC), visual analogue scale (VAS), function and pain subscales of the KSS score. No difference was found in the rate of anterior knee pain, revision, aseptic loosening, fractures, and deep vein thrombosis.

**Conclusion:**

There is no evidence in support that MB implants promote greater outcomes compared to FB implants in primary TKA.

**Level of evidence:**

Level I.

## Introduction

Knee osteoarthritis (OA) is common [6, 94]. Knee OA impairs joint function and quality of life, limiting physical activities and patient independency [71, 73, 115]. Total knee arthroplasty (TKA) is advocated for end-stage knee OA [46, 74, 85]. Both mobile (MB) and fixed (FB) bearing implants are available for primary TKA [1, 49]. FB implants were introduced first, and still represent the most common type of TKA [21, 84]. The polyethylene inlay of FB implants is secured on the tibial plateau. On the other hand, MB implants allow rotation of the polyethylene inlay around its longitudinal axis, miming the physiological kinematics of the knee and promoting a wider range of motion [22, 34, 93]. Previous evidence suggested that MB may promote greater outcomes in functional scores and complications [40, 44, 45, 75, 95]. However, the difference was minimal, and whether mobile bearing provide better outcomes remains controversial [5, 16, 38, 46, 63, 82, 96, 100, 103, 111]. Several randomized clinical trials (RCTs), which have not been yet considered in any previous meta-analyses, have recently been recently published [8, 24, 28, 59, 93, 105, 107, 123]. An update of current evidence could clarify whether MB implants promote greater outcomes to FB in TKA in terms of outcome and complication rate. This meta-analysis compared MB *versus* FB for primary TKA in terms of implant positioning, patient reported outcome measures (PROMs), and complications. It was hypothesised that MB promotes better outcomes than FB implants in primary TKA.

## Materials and methods

### Eligibility criteria

All the clinical investigations comparing mobile *versus* fixed bearing for primary TKA were considered. Only randomized clinical trials (RCTs) with level I to II of evidence, according to Oxford Centre of Evidence-Based Medicine [47], were considered. Only articles in English, German, Italian, French, and Spanish were eligible. Only studies published in peer reviewed journals with accessible full-text article were considered. Only studies which clearly stated the number of included procedures with a minimum of 8 months follow-up were considered. Reviews, opinions, letters, and editorials were not considered. Animals, in vitro, biomechanics, computational, and cadaveric studies were not eligible. All studies investigating the efficacy of experimental rehabilitation protocols were also not included. Studies reporting revision surgeries were also excluded from the analysis.

### Search strategy

This meta-analysis was conducted according to the Preferred Reporting Items for Systematic Reviews and Meta-Analyses: the 2020 PRISMA statement [89]. The PICODT algorithm was preliminary pointed out:

- P (Population): end-stage knee osteoarthritis;
- I (Intervention): TKA;
- C (Comparison): Mb *versus* Fb;
- O (Outcomes): implant alignment, surgical duration, range of motion, PROMs, complications;
- D (Design); RCT;
- T (Follow-up): minimum 8 months.

In February 2022, the following databases were accessed: Pubmed, Web of Science, Google Scholar, Embase. The search was limited to RCTs, with no time constrains. The following keywords were used in combination using the Boolean operator AND/OR: *knee, osteoarthritis, total, arthroplasty, replacement, prosthesis, implant, mobile bearing, fixed bearing, patient reported outcome measures, PROMs, function, efficacy, complication, revision, reoperation, pain, outcome*.

### Selection and data collection

Two authors (F.C. and K.E.) independently performed the database search. All the resulting titles were screened and if suitable, the abstract was accessed. The full-text of the abstracts which matched the topic was accessed. A cross reference of the bibliography of the full-text articles were also screened for inclusion. All disagreements between the authors were debated and, if necessary, solved by a third author (NM).

### Data items

Two authors (F.C. and K.E.) independently performed data extraction.

The following data at baseline were extracted:

- Generalities of the study: name of the first author, year of publication and journal, length of the follow-up, number of patients, percentage of women (%), body mass index (BMI).

The following data at baseline and at last follow-up were extracted:

- Range of motion (ROM);
- PROMs: Oxford Knee Score (OKS), Western Ontario and McMaster Universities Osteoarthritis Index (WOMAC), visual analogue scale (VAS), Knee Society Score (KSS), and relate function (KSFS and pain (KSPS) subscales.
  The following data at last follow-up were collected:
- Implant alignment: tibial slope, delta angle, alpha femoral component angle, gamma femoral component angle, beta tibial component angle, tibiofemoral alignment angle, posterior condylar offset, radiolucent lines;
- Surgical duration;
- Complications: anterior knee pain (AKP), revision, aseptic loosening, fractures, deep vein thrombosis (DVT).

### Study risk of bias assessment

The risk of bias was valuated using the software Review Manager 5.3 (The Nordic Cochrane Collaboration, Copenhagen). The risk of bias was evaluated, based on the guidelines in the Cochrane Handbook for Systematic Reviews of Interventions [27], by the two reviewers (F.C. and K.E.). The following endpoints were evaluated: selection, detection, performance, attrition, reporting, and other bias. To assess the overall risk of publication bias, the funnel plot of the most commonly reported outcome was performed. The funnel plot charted the standard error (SE) of the Log Odd Ratio (Log_OR_) versus its OR. The degree of asymmetry of the plot is directly proportional to the degree of bias. To assess the risk of bias of each included studies, the risk of bias graph was performed.

### Statistical analysis and synthesis methods

The statistical analyses were performed by the main author (F.M.). For descriptive statistics, the IBM SPSS software (version 25) was used. The mean difference and standard deviation were adopted. The *T* test was performed to assess baseline comparability, with values of *P* > 0.1 considered satisfactory. For the meta-analyses, the software Review Manager 5.3 (The Nordic Cochrane Collaboration, Copenhagen) was used. For continuous data, the inverse variance method with mean difference (MD) effect measure was used. For binary data, the Mantel–Haenszel method with odd ratio (OR) effect measure was used. The confidence interval (CI) was set at 0.95 in all the comparison. Heterogeneity was assessed using $\chi$^2^ and Higgins-*I*^2^ tests. If $\chi$^2^ > 0.05, no statistically significant heterogeneity was found. If $\chi$^2^ < 0.05 and Higgins-*I*^2^ > 60% high heterogeneity was found. A fixed model effect was used as default. In case of high heterogeneity, a random model was used. Overall values of *P* < 0.05 were considered statistically significant.

## Results

### Study selection

**Fig. 1:**
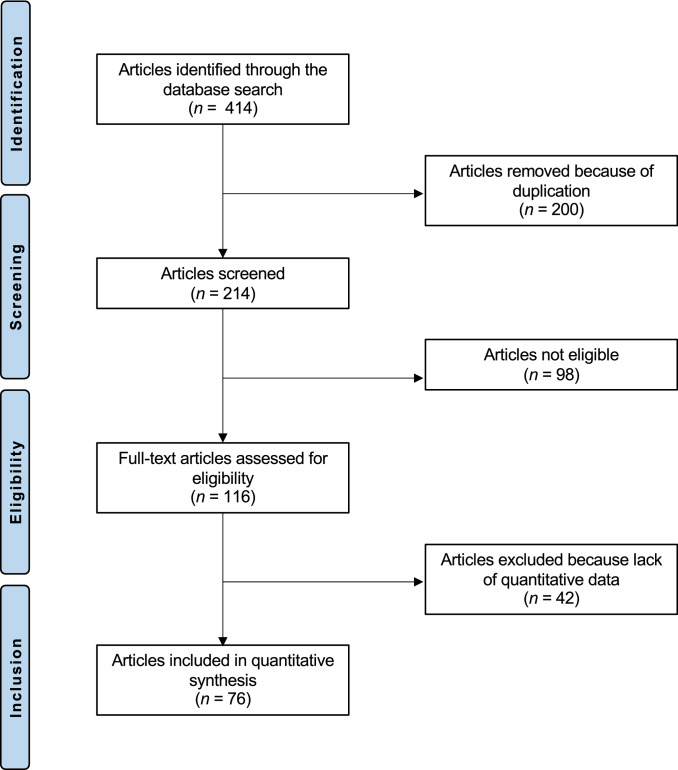
Flow chart of the literature search

The literature search resulted in 414 articles. After removal of duplicates (*N* = 200), a further 140 articles were not eligible for the following reasons: study design (*N* = 78), language limitation (*N* = 17), short follow-up (*N* = 19), lacking quantitative data under the endpoints of interest (*N* = 26). Finally, 74 comparative studies were included. The results of the literature search are shown in Fig. 1.

### Risk of publication bias

**Fig. 2:**
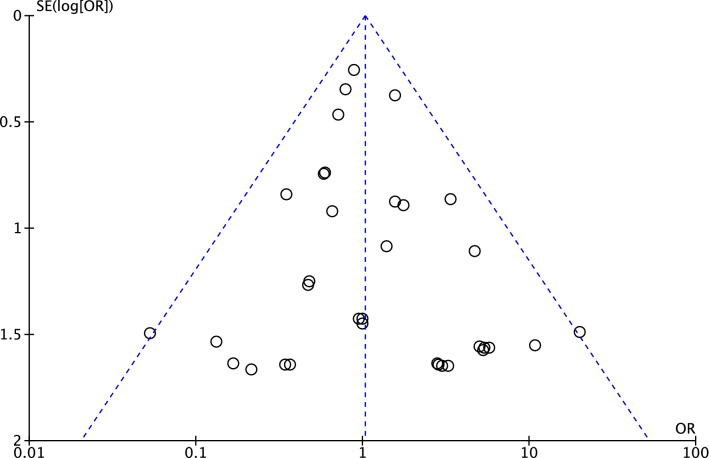
Funnel plot

The funnel plot of the most commonly reported outcome (revision) was performed to assess the risk of publication bias. The plot evidenced very good symmetry, with optimal distribution of the estimated effects of the included studies. The Egger’s test score was *P* = 0.6, attesting a low risk of publication bias (Fig. 2).

### Study risk of bias assessment

**Fig. 3:**
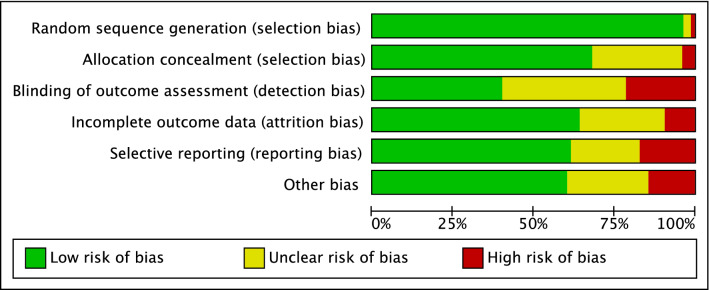
Methodological quality assessment

Given the randomized design of the included studies, the risk of selection bias was low. The risk of detection bias was low to moderate, as was the risk of attrition and reporting biases. The risk of other bias was also low to moderate. Concluding, the quality of the methodological assessment was good. The Cochrane risk of bias graph is shown in Fig. 3.

### Study characteristics and results of individual studies

Data from 11,116 procedures were retrieved. 69% (7670 of 11,116 patients) were women. The mean follow-up was 58.8 (7.5 to 315.6) months. The mean age was 67.5 ± 5.9 years, the mean BMI was 28.6 ± 2.3 kg/m^2^. Comparability was found at baseline concerning the mean age, mean BMI, female, ROM, KSS, OKS, KSS pain, WOMAC, VAS, and KSS function. Generalities and patient baseline of the included studies are shown in greater detail in Table 1, the baseline comparability between the two groups at baseline in Table 2.

**Table 1:** Generalities and patient baseline of the included studies

Author, year	Journal	Follow-up (months)	Bearing	Procedures (*n*)	Mean age	Mean BMI	Women (%)
Abdel et al., 2018 [2]	Bone Joint J	120.1	APE FB	50	67		
			Metal backed FB	66	67		
			MB	53	67		
Aggarwal et al., 2013 [4]	J Arthroplasty	66	MB	29	60	27.4	83
			FB	27	54.6	25.3	85
Aglietti et al., 2005 [5]	J Arthroplasty	36	MB	103	71	27.5	86
			FB	107	69.5	27.5	81
Amaro et al., 2016 [7]	Knee Surg Sports Tramatol Arthrosoc	24	FB	32	66.2	29.8	69
			MB	32	65.2	31.1	75
Amaro et al., 2019 [8]	J Knee Surg	24	FB	32	66.2	29.8	69
			MB	32	65.2	31.1	75
Artz et al., 2015 [9]	J Arthroplasty	24	FB	102	61.6		47
			MB	104	61.7		55
Bailey et al., 2014 [10]	Knee Surg Sports Tramatol Arthrosoc	24	MB	161	69.2	30.4	69
			FB	170	70.1	31.6	70
Baktir et al., 2016 [11]	Acta Orthop Traumatol Turc	72	MB	47	64.9	33.3	87
			FB	46	64.7	32.2	89
Ball et al., 2011 [12]	J Arthroplasty	48	MB	51	64.9	31.0	56
			FB	42	64.0	31.0	56
Beard et al., 2007 [13]	Knee	36	MB	33	73.1		60
			FB	33	73.1		60
Bhan et al., 2005 [14]	J Bone Joint Surg	54	FB	32	63		69
			MB	32	63		69
Breeman et al., 2013 [17]	Bone Joint J	60	MB	276	69	29.5	16
			FB	263	69	30.3	59
Breugrem et al., 2008 [18]	Clin Orthop Relat Res	12	FB	53	68.9	29.1	64
			MB	47	71.2	28.4	65
Breugem et al., 2012 [19]	Knee Surg Sports Traumatol Arthrosc	94.8	FB	40	80		65
			MB	29	78		65
Chaudhry et al., 2018 [23]	J Orthop Traumatol	90	FB	60	57.6	25.1	72
			MB	50	58.7	25.7	71
Choi et al., 2010 [25]	J Bone Joint Surg	24	MB	85	70.1	26.6	93
			FB	85	71.1	26.5	97
Feczko et al., 2017 [31]	BMC Musculoskelet Disord	60	FB	48		30.1	
			MB	42		28.7	
Ferguson et al., 2014 [32]	Knee	24	MB	163	69.8	29.7	53
			FB	163	70.2	31.1	53
Fransen et al., 2015 [33]	J Arthroplasty	72	MB	77	65.7	30.2	68
			FB	69	63.8	30.2	72
Garling et al., 2005 [35]	Acta Orthop	24	MB	21	66	27.0	50
			FB	21			
Gioe et al., 2009 [36]	J Bone Joint Surg	24	MB	176	71.79	31.9	2
			FB	136	72.62	31.5	4
Hansson et al., 2005 [38]	Knee	24	MB	25	74		48
			FB	27	75		52
Hanusch et al., 2010 [39]	Int Orthop	24	FB	55	69.4	29.9	40
			MB	50	70	29.7	60
Harrington et al., 2009 [41]	J Arthroplasty	24	FB	72	63.3	34.2	69
			MB	68	63.7	34.2	59
Hasegawa et al., 2008 [42]	Knee Surg Sports Traumatol Arthrosc	40	MB	25	73	25.2	88
			FB	25	73	25.2	88
Henricson et al., 2006 [43]	Clin Orthop Relat Res	24	MB	26	72		
			FB	26	72		
Jacobs et al., 2011 [48]	Knee Surg Sports Traumatol Arthrosc	12	MB	46	67.6		71
			FB	46	66.7		70
Jolles et al., 2012 [50]	J Bone Joint Surg	60	MB	26	67.1	29.6	68
			FB	29	70.2	27.9	48
Kalisvaart et al., 2012 [51]	J Bone Joint Surg	60	FB (polyethylene)	75	67	32.1	69
			FB (modular-metal-backed)	76	67.1	30.5	70
			MB	76	67.4	33.1	70
Kim et al., 2007 [56]	J Bone Joint Surg	67.2	MB	174	67	26.7	64
			FB	174	67	26.7	64
Kim et al., 2007 [64]	J Bone Joint Surg	158.4	FB	146	69.8	27.5	94
			MB	146	69.8	27.5	94
Kim et al., 2008 [63]	Clin Orthop Relat Res	24	FB	92	69.5	27.8	92
			MB	92	69.5	27.8	92
Kim et al., 2009 [57]	J Arthroplasty	24	FB	61	48.3	26.8	74
			MB	61	48.3	26.8	74
Kim et al., 2009 [55]	Knee Surg Sports Traumatol Arthrosc	24	FB	66	70	26.0	97
			MB	66	70	26.0	97
Kim et al., 2011 [54]	Knee Surg Sports Traumatol Arthrosc	30	MB	37	68	27.3	95
			FB	36	66	27.1	98
Kim et al., 2017 [60]	J Arthroplasty	134.4	FB	92	61.5	26.2	82
			MB	92	61.5	26.2	82
Kim et al., 2012 [58]	J Bone Joint Surg	201.6	MB	108	45	25.6	77
			FB	108	45	25.6	77
Kim et al., 2014 [62]	J Bone Joint Surg	144	MB	444	66.5	29.6	93
			FB	444	66.5	29.6	93
Kim et al., 2018 [61]	J Arthroplasty	156	MB	164	63	28.0	87
			FB	164	63	28.0	87
Kim et al., 2020 [59]	J Arthroplasty	315.6	MB	291	58	27.0	77
			FB	291	58	27.0	77
Killen et al., 2019 [53]	J Clin Orthop Trauma	144	FB	19	76.79		76
			MB	28	76.57		60
Lädermann et al., 2007 [66]	Knee	36	FB	52	79	29.9	77
			MB	50	72	29.6	60
Lädermann et al., 2008 [67]	Rev. Chir. Orthop. Reparatrice Appar. Mot	85.2	FB	48	69.8	29.9	77
			MB	44	72	29.6	60
Lizaur-Utrilla et al., 2012 [70]	J Arthroplasty	24	MB	61	74.6	31.3	77
			FB	58	73.9	32.6	81
Mahoney et al., 2012 [72]	Clin Orthop Relat Res	24	MB	178	66	31.0	67
			FB	183	66	31.0	61
Marques et al., 2014 [75]	Knee Surg Sports Traumatol Arthrosc	48	FB	45	68.9	28.7	75
			MB	42	69.4	30.4	70
Matsuda et al., 2010 [76]	Knee Surg Sports Traumatol Arthrosc	70.8	FB	31	76		78
			MB	30	73		77
Minoda et al., 2014 [83]	Knee Surg Sports Traumatol Arthrosc	24	MB	46	74.3	26.3	89
			FB	48	75.7	25.5	87
Niuewenhuijse et al., 2013 [83]	J Bone Joint Surg	70	LPS-Flex MB	16	66.8	25.9	79
			LPS-Flex FB	12	72.2	26.5	70
			LPS MB	14	68.7	29.0	100
			LPS FB	19	68.5	27.6	76
Nutton et al., 2012 [87]	J Bone Joint Surg	12	FB	40	69.8	29.8	53
			MB	36	68.3	29.1	50
Okamoto et al., 2014 [88]	J Arthroplasty	12	MB	20	76	25.0	90
			FB	20	78	27.0	80
Park et al., 2018 [91]	Knee Surg Sports Traumatol Arthrosc	24	MB	70	69.5	26.0	93
			FB	70	68.9	25.6	96
Pijls et al., 2012 [92]	J Bone Joint Surg	120	MB	21	64	27.0	86
			FB	21	66	27.0	76
Poirier et al., 2015 [93]	Orthop Traumatol Surg Res	108	FB	31	72		58
			MB	30	70		53
Powell et al., 2018 [95]	Bone Joint J	60	MB	46	65.5	29.7	44
			FB	39	65.5	29.7	44
Price et al., 2003 [96]	J Bone Joint Surg	12	FB	19	73.1		60
			MB	21	73.1		60
Radetzki et al., 2013 [97]	Acta Orthop	120	FB	22	65.5	24.4	60
			MB	17	66.5	24.1	53
Rahman et al., 2010 [98]	J Arthroplasty	43	MB	24	62.6	31.5	58
			FB	27	62	31.4	67
Roh et al., 2012 [102]	Knee Surg Sports Traumatol Arthrosc	30	MB	42	69.8	26.5	95
			MB	44	71	26.4	93
Sappey-Marinier et al., 2019 [104]	Knee Surg Sports Traumatol Arthrosc	60	FB	64	71	29.0	58
			MB	65	71	30.0	60
Sappey-Marinier et al., 2020 [105]	Knee Surg Sports Traumatol Arthrosc	120	FB	50	71	29.0	58
			MB	56	71	30.0	60
Schotanus et al., 2016 [106]	Knee Surg Sports Traumatol Arthrosc	24	MB	20	62.7	29	48
			FB	22	67.3	29.4	41
Schotanus et al., 2017 [107]	Eur J Orthop Surg Traumatol	24	MB	20	61.9	29.4	40
			FB	21	67.1	29.9	43
Scuderi et al., 2012 [109]	J Arthroplasty	48	MB	152	63.7	29.6	55
			FB	141	63.4	29.4	62
Shemanski et al., 2012 [110]	Knee Surg Sports Traumatol Arthrosc	72	FB	150	70		68
			MB	150	68		60
Tiwari et al., 2019 [113]	Knee Surg Sports Traumatol Arthrosc	24	MB	260	69.7	26.9	94
			FB	133	69.7	26.7	98
Tjornild et al., 2015 [114]	Acta Orthop	24	FB	23	66	30.0	46
			MB	23	66	27.0	65
Urwin et al., 2014 [116]	Knee	9	FB	8	59.3	31.9	38
			MB	8	59.6	31.9	38
Van hammersfeld et al., 2018 [117]	Acta Orthop	72	FB	16	68	30.1	70
			MB	12	67.5	29.8	83
Vasdev et al., 2009 [118]	J Orthop Surg		FB	60	63		67
		42	MB	60	63		50
Watanabe et al., 2005 [119]	Int Orthop	96	MB	22	59.6		96
Wohlrab et al., 2005 [120]	Z Orthop	35	FB	30	65,5	24.4	62
			MB	30	66,5	24.1	53
			FB	22	59.6		95
Woolson et al., 2011 [121]	J Arthroplasty	120	FB	30	77.9	29.2	
			MB	31	78	27.7	
Wylde et al., 2008 [122]	J Bone Joint Surg	24	FB	120	67.6		64
			MB	108	68.9		68

MB: mobile bearing; FB: fixed bearing

**Table 2:** Baseline comparability of the two groups

Endpoint	FB (*n* = 5517)	MB (*n* = 5599)	*P *values
Mean age	67.5 ± 61	67.3 ± 5.6	n. s
Mean BMI	28.7 ± 2.3	28.6 ± 2.3	n. s
Women (%)	1.7 ± 8.9	1.5 ± 6.9	n. s
ROM	104.9 ± 24.5	105.0 ± 24.1	n. s
KSS	39.7 ± 17.0	40.5 ± 17.1	n. s
OKS	33.1 ± 10.9	33.3 ± 10.9	n. s
KSS pain	25.3 ± 26.5	21.1 ± 25.7	n. s
WOMAC	59.9 ± 8.5	59.2 ± 8.3	n. s
VAS	32.8 ± 36.0	32.5 ± 33.0	n. s
KSS function	43.3 ± 12.8	43.5 ± 12.7	n. s

No statistically significant difference was detected

MB: mobile bearing; FB: fixed bearing; MD: mean difference; ROM: range of motion; OKS: Oxford Knee Score; WOMAC: Western Ontario and McMaster Universities Osteoarthritis Index; VAS: visual analogue scale; KSS: Knee Society Score; n. s.: not significant

### Results of syntheses

**Fig. 4:**
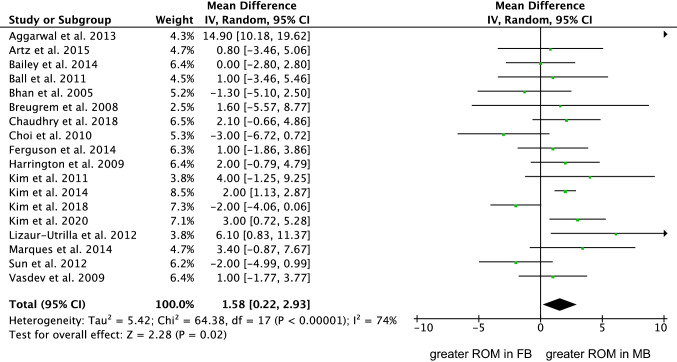
Forest plot of the comparison: ROM (IV: inverse variance; CI: confidence interval). The square represents the effect of each single study. The horizontal line represents the confidence interval of each study. The vertical line “0” represent the no effect threshold. The diamond represents the final effect of the overall analysis

Eighteen studies (3827 procedures) were included in the comparison of ROM [4, 9, 10, 12, 14, 18, 23, 25, 41, 54, 59, 60, 62, 70, 75, 118]. The MB group demonstrated greater ROM (MD 1.58; 95% CI 0.22 to 2.93; *P* = 0.02; Fig. 4).

**Fig. 5:**
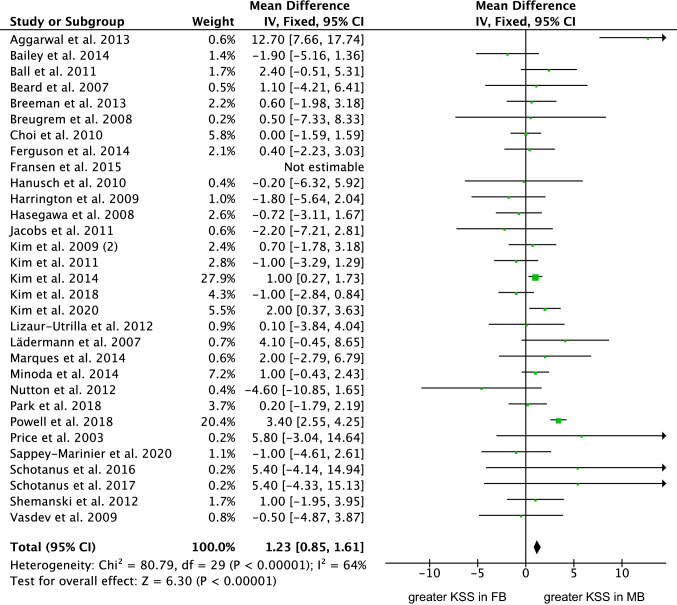
Forest plot of the comparison: KSS score (IV: inverse variance; CI: confidence interval). The square represents the effect of each single study. The horizontal line represents the confidence interval of each study. The vertical line “0” represent the no effect threshold. The diamond represents the final effect of the overall analysis

Thirty-one studies (5094 procedures) were included in the comparison of the KSS score [4, 10, 12, 13, 17, 18, 25, 32, 33, 39, 41, 42, 48, 54, 57, 59, 60, 62, 66, 70, 75, 83, 87, 91, 95, 96, 104, 106, 107, 110, 118]. The MB evidenced greater KSS score (MD 1.23; 95% CI 0.85 to 1.61; *P* < 0.0001; Fig. 5).

**Fig. 6:**
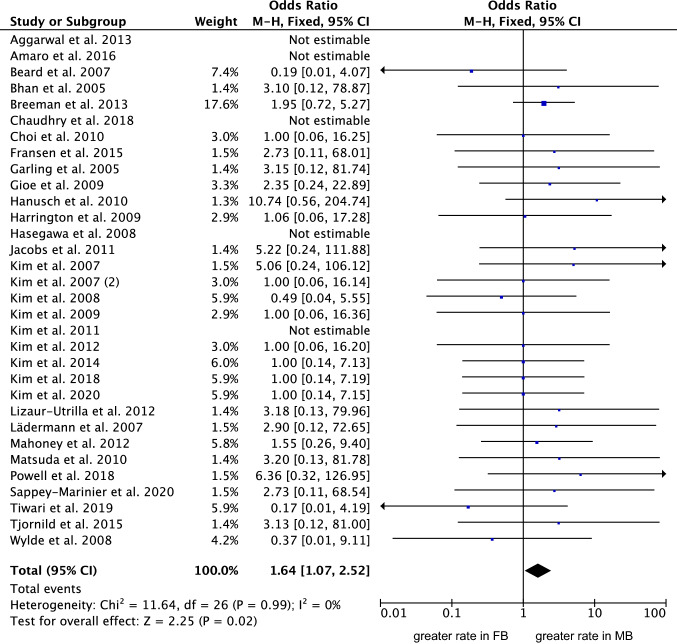
Forest plot of the comparison: rate of deep infection (M–H: Mantel–Haenszel; CI: confidence interval). The square represents the effect of each single study. The horizontal line represents the confidence interval of each study. The vertical line “0” represent the no effect threshold. The diamond represents the final effect of the overall analysis

Thirty-two studies (6489 procedures) were included in the comparison of rate of deep infection [4, 7, 13, 14, 17, 23, 25, 33, 35, 36, 39, 41, 42, 55–57, 60–64, 67, 70, 72, 76, 95, 104, 113, 122].The MB group evidenced a greater rate of deep infections (OR 1.64; 95% CI 1.07 to 2.52; *P* = 0.02; Fig. 6).

No difference was found in implant positioning: tibial slope, delta angle, alpha femoral component angle, gamma femoral component angle, beta tibial component angle, tibiofemoral alignment angle, posterior condylar offset, radiolucent lines. No difference was found in duration of the surgical procedure. No differences were found in the following PROMs: OKS, WOMAC, VAS, function and pain subscales of the KSS score. No difference were found in the rate of the following complications: AKP, revision, aseptic loosening, fractures, DVT.

## Discussion

The main finding of the present study was that the MB implants performed in a similar fashion to FB implants for TKA. The analyses evidenced greater KSS, ROM, and rate of the deep infection in MB implants. However, though statistically significant, their clinical relevance is likely limited. Concerning the KSS score, its overall difference between the two implants does not overcome their minimal clinically important difference (MCID), which has been estimated between 6/100 and 9/100 [40, 52, 68, 69, 112]. A formal MCID for the ROM has not yet been estimated. However, given its minimal difference, the clinical relevance of this finding is dubious. Marques et al. [75] conducted a RCT on 99 patients. After 1-year follow-up, a statistically significant increase in ROM and KSS was found in the MB group. After 4-year follow-up no difference was found between the MB and FB group. Kim et al. [59] conducted a RTC on 291 patients, with a follow-up period of 27 years. No differences were found in ROM and KSS between the two groups. Powell et al. [95] analysed 167 patients at 10 years of follow-up, with no statistically significant difference in KSS between MB and FB groups. However, a trend was seen with higher mean scores over the years for the MB group. Given the minimal difference between the two groups in ROM and KSS, the clinical relevance of these findings was dubious. A slight improvement of PROMs was not necessarily associated with a functional advantage [15]. The minimal functional improvement may be explained by greater axial rotation promoted by the MB implants [29, 53, 99]. Amaro et al. [8] evaluating kinematic differences in 64 patients, found that axial rotation was higher in the MB group after 1 year, but disappeared at 2-year follow-up. A histological study showed the development of fibrotic tissue in the synovial membrane and infrapatellar fat pad after a TKA [3]. This produces a hardening effect that may minimize the kinematic differences between the MB and FB groups [8]. MB actively corrects the rotational femoral offset while standing, improving stepping and squatting [46]. However, this difference is not clinically relevant [88]. Moreover, different types of MB implants have different kinematics during stepping and squatting [46], and the final clinical outcome of MB can be influenced by the brand. A long-term study comparing different types of MB and FB implants could be useful to further understand the real benefits of different type of prostheses.

The rate of deep infection was strongly influenced by the study by Breeman et al. [17], which weighted 17.6% on the final effect. Indeed, when conducting the analyses without those data [17], the rate of deep infection is similar between the two groups. Nevertheless, the authors evidenced no difference between the two implants in terms of infections in their study [17]. Indeed, a deep infection was present in 12 of 276 patients in the MB group, and in 6 of 263 patients in the FB group [17]. Some limitations that may have influence our results should be discussed. The authors conducted a multicentre study involving 116 surgeons [17, 99]. Surgeon experience and approaches, implants design and post-operative protocols were not considered.

No differences in radiographic alignment were shown in the present study. Only one study showed a radiographic difference in patellar translation [104]. A tendency to increase patellar translation in the MB group was also evidenced in the present study. In MB implants, the rotation of the tibial component and the variable position of the tibial relative to the femoral implant can affect patellar tracking [90]. However, other meta-analyses comparing patellar translation did not evidence any differences between MB and FB implants [86, 111].

The MB design has been introduced to better simulate knee kinematics, reducing contact stresses, aseptic loosening, and polyethylene wear [20]. The self-alignment promoted by the MB implants compensates the physiological tibial and the femoral component offset [30]. The latter has been hypothesized to improve the conformity between femoral component and mobile insert during stepping and squatting, thus reducing contact pressure and loosening of polyethylene wear [46]. However, this study was unable to identify differences between the two implants, in contest with previous evidence [59, 62, 105]. Though there is less wear at the femoral condyle interface in MB than in FB implant, the former produce additional wear at the surface of metallic tibial implant, which may explain the similarity in the rate of overall wear [105]. Only one study [37] showed a higher rate of aseptic loosening in the MB group. The risk was higher only in certain models. In the MB implants, the geometry of tibial component is such that the shortening of the keel and the under-face texture increase the risk for micromotion and aseptic loosening [26, 53, 65, 101, 104, 108].

This study certainly has limitations. The analyses were conducted irrespective of the surgical exposure and approach. In the present study, both minimally and standard invasive techniques were included. Surgical exposure may influence outcomes, and minimally invasive surgery performed by experienced surgeon may offer short- and mid-term clinical and functional benefits over the conventional exposure [78]. Moreover, the surgical approach may influence the clinical outcomes. A recent network meta-analysis demonstrated that the mini-subvastus approach outperformed all other approaches (mini-medial parapatellar, midvastus, quadriceps sparring) [77]. Patellar retaining or resurfacing has not been investigated, and may represent a further limitation [80]. Different inlay designs (posterior stabilized, cruciate/bicruciate retaining) were not considered as separate. A previous meta-analysis demonstrated no difference in the outcome between the posterior stabilized versus cruciate retaining [81], while no study which compared MB versus FB using bicruciate retaining implants were included in the present study. The manufacturer of the implants was often biased. MB implants are more sensitive to soft tissue release and optimal gap balancing over flexion and extension. Differently, in FB implants planned resection following the anatomical landmarks (anteroposterior and trans-epicondylar axis) can be performed [79]. Few authors appropriately described the surgical protocol, and further subgroups comparisons were not possible. This may generate bias and increase heterogeneity. The conclusion of the present meta-analysis should be considered with these limitations. Results of the present study indicated that bearing in TKA, whether mobile or fixed, does not influence the clinical outcome.

## Conclusion

There is no evidence to support that MP implants promote better outcomes compared to FB implants in primary TKA. The analyses evidenced greater KSS, ROM, and greater rate of the deep infection in MB implants. However, though statistically significant, their clinical relevance is limited. Further clinical trials are required.

## Data Availability

The datasets generated during and/or analysed during the current study are available from the corresponding author on reasonable request.
